# Correlation Between Air Quality and the Exacerbation of Asthma Symptoms in the Population of the United Arab Emirates

**DOI:** 10.7759/cureus.93212

**Published:** 2025-09-25

**Authors:** Iman Hagana, Sarah Choudhry, Maria E Iacob, Mary Ann Boniface, Aadil Ashraf Ahmed Shaikh, Syed Arshad Husain

**Affiliations:** 1 Internal Medicine, Ipswich Hospital, East Suffolk and North Essex NHS Trust, Ipswich, GBR; 2 Emergency Medicine, Royal Berkshire Hospital, Reading, GBR; 3 Transplant Surgery, Addenbrooke's Hospital, Cambridge University Hospitals NHS Foundation Trust, Cambridge, GBR; 4 Intensive Care Unit, Mercy Health Fairfield Hospital, Ohio, USA; 5 Internal Medicine, Wellstar Cobb Medical Center, Austell, USA; 6 Pulmonary Medicine, Al Zahra Hospital, Dubai, ARE; 7 Pulmonary Medicine, Gulf Medical University, Dubai, ARE

**Keywords:** air quality, asthma, particulate matter, pulmonology, united arab emirates

## Abstract

Background

Asthma is a chronic respiratory disease, characterised by shortness of breath (SOB), wheeze, cough and chest tightness, that has a significant impact on quality of life and global health. Previous studies have demonstrated that environmental pollutants, particularly particulate matter (PM_2.5_), play a key role in triggering and exacerbating asthma. Despite high atmospheric air pollution in the United Arab Emirates (UAE), there is limited region-specific research evaluating its effect on asthma symptoms. This study aimed to investigate the association between daily PM_2.5_ levels and patient-reported asthma symptoms in adults residing in Dubai, UAE.

Methods

A retrospective observational study conducted between April to June 2024, using 284 outpatient consultation records from pulmonology clinics at a single-centre, King’s College Hospital London in Dubai, UAE. Inclusion criteria involved adults aged 18 and above, a formal diagnosis of asthma, and a minimum of one year of residency in Dubai prior to the study period. Symptoms, including SOB, cough, wheeze, and chest tightness, were extracted from electronic medical records. These symptoms were matched to daily PM_2.5_ data obtained from an online air quality server, known as IQAir. Associations were assessed using chi-square tests and logistic regression, adjusting for age, sex, body mass index (BMI), smoking status, allergy status, pet ownership, and consultation type.

Results

In the final analysis, 284 consultations were included. From these, 162 were male (57%) and 122 were female (43%) participants. SOB (60.9%) and cough (46.1%) were the most frequently reported symptoms. Logistic regression revealed that higher PM_2.5_ categories (“Unhealthy for sensitive groups”) were associated with a lower likelihood of reporting SOB (adjusted OR 0.53, 95% CI 0.29-0.98, p=0.045). No statistically significant associations were found between PM_2.5 _and cough, wheeze, or chest tightness. Age was independently associated with SOB, with the youngest group (18-29 years) having a higher incidence of SOB compared to older age groups.

Conclusions

Contrary to established evidence, higher PM_2.5_ levels were inversely associated with SOB among asthma patients in the UAE. This unexpected association should not be interpreted as suggesting a protective role of air pollution; rather, it is more plausibly explained by numerous factors, including methodological constraints such as consultation-related confounding, or patient factors, including behavioural avoidance. Furthermore, given that these results diverge from prior research, the limited sample size and statistical power should be considered when interpreting the findings. As the first pilot study in the UAE linking daily air quality with clinical asthma outcomes, it is evident that larger, long-term studies incorporating seasonal variations and additional pollutants are necessary.

## Introduction

Asthma is a common chronic respiratory disease, characterised by wheeze, shortness of breath (SOB), chest tightness, and cough, as a result of chronic airway inflammation as per the Global Initiative for Asthma (GINA) [1]. According to the World Health Organisation (WHO), in 2019, asthma affected approximately 262 million people globally, with 455,000 deaths [2]. In the United Arab Emirates (UAE), although there is limited data, recent reports estimate 7.4% of the UAE population suffers from asthma [3], with the global burden of disease estimating 8.1% in 2021 [4]. Asthma significantly impacts quality of life, with psychological, medical and financial burdens, for both sufferers and their families [5].

It is widely recognised that asthma is a complex condition, with unclear aetiology, but undoubtedly influenced by the interaction of genetic, environmental, and immunological factors. While genetic predisposition plays a role in the development of asthma, environmental exposures, particularly to air pollution, are now widely recognised as key contributors to both the onset and exacerbation of the disease [6]. Air pollutants such as particulate matter (PM_2.5_ and PM_10_), nitrogen dioxide (NO_2_), sulfur dioxide (SO_2_), ozone (O_3_), and carbon monoxide (CO) can trigger airway inflammation, bronchial hyperresponsiveness, and oxidative stress, all of which are central to asthma pathophysiology [7]. Fine particulate matter (PM_2.5_) is especially harmful due to its ability to penetrate deep into the lungs, leading to systemic inflammation and increased sensitivity to allergens.

Numerous epidemiological studies have shown that short- and long-term exposure to polluted air is associated with increased asthma symptoms, admissions to the emergency department, and even the development of asthma in children and adults [8]. However, in urban settings, such as the UAE, where rapid industrialisation, significant vehicular emissions, and frequent dust exposure contribute to poor air quality, there is limited region-specific research examining how these climatic conditions in the UAE impact asthma severity.

This study aims to fill this gap by assessing the impact of air pollution on asthma in the population of Dubai, UAE, through evaluating the association between daily PM_2.5_ levels and patient-reported asthma symptoms. By analysing governmental air quality data and reported daily asthma symptoms collected on specific days, this research seeks to provide valuable evidence on how environmental factors influence asthma outcomes in this setting. Given the known effects of PM_2.5_ on airway inflammation [7], it is expected that higher PM_2.5_ levels would be associated with an increase in asthma symptoms. The findings could potentially contribute to the development of public health interventions and environmental policies aimed at mitigating asthma morbidity in the UAE.

## Materials and methods

Study design and setting

This is a retrospective observational study designed to assess the correlation between atmospheric conditions and the severity of asthma symptoms in adults residing in Dubai, UAE. The study was conducted using patient data from the Pulmonology Outpatient Clinic at King's College Hospital London in Dubai, alongside publicly available air quality data collected from IQAir [9].

Ethical approval

All data used was anonymised prior to analysis to ensure patient confidentiality. Ethical approval for this retrospective study was obtained from the Research Ethics Committee, King's College Hospital London in Dubai (KCH/MOI/740, dated April 5, 2024) in accordance with the UAE guidelines. 

Study population

The study population consisted of adult patients aged 18 years and older with a formal clinical diagnosis of asthma. To ensure consistency in environmental exposure, inclusion criteria required participants to have resided in Dubai for at least one year prior to the study period. Patients under the age of 18, without a formal asthma diagnosis or those who had lived in Dubai for less than one year were excluded from the analysis. Eligible participants were identified through the electronic medical record (EMR) system from the pulmonology outpatient clinic.

Data collection

Data was collected retrospectively for the period from April 23, 2024, to June 14, 2024. Two primary data sources were used, including patient symptom data and air quality data. Symptoms (cough, SOB, chest tightness, wheeze) of patients attending the pulmonology outpatient clinic were obtained from the electronic medical record EMR system (Cerner Corporation, Kansas City, MO, USA) from the physician’s notes. Documentation followed a general outpatient structure depending on the consultation type, providing some consistency across consultations. However, there was no formal framework, with symptom presence coded as binary variables (present/absent) based on explicit mention in the notes. The dataset included 'new' patient consultations, 'symptomatic' individuals visiting the clinic and routine 'follow-up' consultations, meaning that some patients contributed more than one visit during the study period.

This data related to the patient's symptoms was extracted on the dates of patient visits within the specified study period. Daily air pollution data were obtained from IQAir, including air quality index (AQI) and PM_2.5 _[9]. IQAir reports daily averages derived from hourly monitoring data aggregated from 56 outdoor air quality stations across Dubai, listed by IQAir​​​​. Days with incomplete air pollution data were excluded from the analysis, ensuring consistency across the study period. The same-day average of AQI and PM_2.5 _was matched to the corresponding dates of patient clinic visits.

Statistical analysis

Complete-case analysis was performed using RStudio 4.3.2 (RStudio, PBC, Boston, MA, USA). For the purpose of complete-case analysis, records with missing values in any variables used in modelling were excluded. Descriptive analysis was performed for baseline demographic data such as sex, age group, body mass index (BMI), then for the exposure PM_2.5_ and for the outcomes of reported SOB, cough, wheeze and chest tightness. Cross-tabulations of the main demographic data against the exposure and outcomes of interest were performed along with chi-square tests to find possible associations and identify possible confounding. To improve model stability and reduce collinearity risk, certain categorical variables with sparse subgroups were collapsed: ‘Underweight’ and ‘Normal’ into a single BMI category, and ‘Good’ with ‘Moderate’ PM_2.5_ levels into one air quality category.

Finally, logistic regression was used to explore the association between air quality and the outcomes of interest, controlling for the possible confounders. Final models were adjusted for age group, sex, BMI category, smoking status, allergy status, pet ownership and consultation type (new, symptomatic, follow-up). These adjustment variables were chosen based on the available collected data, and among those, the variables that could be reasonably associated with the outcome were kept in the model. The Wald test was used to analyse the P-value.

## Results

Consultations occurred between April 23, 2024 and June 14, 2024, for a total of 36 days. The sample included 284 individual consultation records. Table 1 demonstrates the demographic data for the population sample, a total of 284 patients.

**Table 1:** Demographic details of the sample population attending pulmonology outpatient clinics at King's College Hospital London in Dubai BMI: Body Mass Index

Demographic	Number of patients (%)
Age (years)	
18-29	48 (16.9)
30-39	102 (35.9)
40-49	67 (23.6)
50-59	37 (13.0)
60+	30 (10.6)
Sex	
Male	162 (57.0)
Female	122 (43.0)
BMI (kg/m^2^)	
Underweight	4 (1.4)
Normal weight	118 (41.5)
Overweight	109 (38.4)
Obese	53 (18.7)
Smoking	
Current smoker	45 (15.8)
Ex-smoker	25 (8.8)
Non-smoker	214 (75.4)
Pet ownership	
Yes	57 (20.1)
No	227 (79.9)
Allergy status	
Yes	76 (26.8)
No	208 (73.2)

Three consultations occurred when PM_2.5_ was ‘Good’, 182 when it was ‘Moderate’, 80 when it was ‘Unhealthy for sensitive groups’ and 19 when it was ‘Unhealthy’. SOB and cough were the most reported symptoms, being present in 60.9% and 46.1% of patients, respectively. 30.3% of patients complained of chest tightness, and 21.1% of wheeze.

On cross tabulation and chi-square tests, there was a statistically significant association between PM_2.5_ level and the reporting of SOB (p=0.03). There was no significant association on chi-square tests between PM_2.5_ level and the remaining outcomes of cough, wheeze and chest tightness. 'Consultation Type' was associated with all four outcomes with p<0.001. SOB was associated with age group and allergy status (p<0.05 and p<0.04, respectively). Cough was associated with age (p=0.02).

Results of the logistic regression models are reported in Table 2. Logistic regression revealed that the odds ratio of reporting SOB when comparing exposure to ‘Unhealthy for sensitive groups’ and ‘Unhealthy’ PM_2.5_ vs ‘Good/Moderate’ PM_2.5_ were 0.60 (95% CI 0.35 - 1.02, p=0.06) and 0.34 (95% CI 0.11 - 0.92, p=0.045) respectively. Once adjusted, OR was 0.53 (95% CI 0.29 - 0.98) and 0.33 (95% CI 0.09 - 1.04). With increasing levels of PM_2.5_, there was a lower likelihood of reporting SOB. Logistic regression models for the association between PM_2.5_ and the remaining symptoms of cough, chest tightness and wheeze did not show any statistically significant results in the unadjusted and adjusted phases.

**Table 2:** Association between PM₂.₅ and symptoms of SOB, cough, chest tightness, wheeze in unadjusted and adjusted* logistic regression models (reference = good/moderate PM₂.₅) * adjusted for age group, sex, BMI, smoking status, allergy status, and pets P-value analysed using the Wald test PM: particulate matter, SOB: shortness of breath, CI: confidence interval, OR: odds ratio

Symptom	PM_2.5 _exposure	OR (95% CI)		P-value (p)	
		Unadjusted	Adjusted	Unadjusted	Adjusted
SOB	Unhealthy for sensitive groups	0.60 (0.35-1.02)	0.53 (0.29-0.98)	0.060	0.045
	Unhealthy	0.34 (0.11-0.92)	0.33 (0.09-1.04)	0.045	0.071
Cough	Unhealthy for sensitive groups	0.89 (0.52-1.53)	0.83 (0.43-1.58)	0.678	0.562
	Unhealthy	0.54 (0.2-1.39)	0.53 (0.16-1.69)	0.197	0.286
Chest tightness	Unhealthy for sensitive groups	0.96 (0.54-1.69)	0.95 (0.48-1.83)	0.895	0.879
	Unhealthy	0.80 (0.25-2.21)	0.67 (0.17-2.29)	0.685	0.541
Wheeze	Unhealthy for sensitive groups	1.09 (0.57-2.02)	0.93 (0.43-1.94)	0.796	0.851
	Unhealthy	0.70 (0.16-2.24)	0.64 (0.12-2.54)	0.589	0.548

Table 3 shows the adjusted model for the association between PM_2.5_ exposure and SOB. Significant association was found between allergy status and SOB, whereby patients with allergy were less likely to report the symptom (OR 0.51, 95% CI 0.27 - 0.95, p=0.037). When compared to the youngest age group, 18-29, older age groups were less likely to report SOB. Patients attending ‘Follow-up’ consultations were likely to report SOB when compared to ‘New’ consultations (OR=0.20, 95% CI 0.10 - 0.37, p <0.001). No evidence of multicollinearity was found among the included variables.

**Table 3:** Adjusted logistic regression model for the association between SOB and PM₂.₅ exposure P-value analysed using the Wald Test Ref: reference; PM: particulate matter; SOB: shortness of breath; OR: odds ratio; CI: confidence interval; BMI: Body Mass Index

Variable	Adjusted OR	95% CI	P-value (p)
PM_2.5_ exposure			
Good/moderate (ref)	ref	ref	ref
Unhealthy for sensitive groups	0.53	0.29 – 0.98	0.045
Unhealthy	0.33	0.09 – 1.04	0.071
Age (years)			
18–29 (ref)	ref	ref	ref
30–39	0.52	0.23 – 1.13	0.120
40–49	0.36	0.15 – 0.84	0.020
50–59	0.33	0.12 – 0.88	0.029
60+	0.16	0.05 – 0.50	0.002
Sex			
Female (ref)	ref	ref	ref
Male	0.64	0.36 – 1.13	0.122
BMI category (kg/m^2^)			
Underweight/normal (<25) (ref)	ref	ref	ref
Overweight (25-29.9)	1.10	0.60 – 2.02	0.760
Obese (>30)	0.94	0.43 – 2.05	0.873
Smoking status			
Non-smoker (ref)	ref	ref	ref
Current smoker	2.54	0.96 – 7.14	0.067
Ex-smoker	0.68	0.31 – 1.46	0.322
Pet ownership			
No (ref)	ref	ref	ref
Yes	1.34	0.68 – 2.66	0.394
Allergy status			
No (ref)	ref	ref	ref
Yes	0.51	0.27 – 0.95	0.037
Consultation type			
New (ref)	ref	ref	ref
Follow-up	0.20	0.10 – 0.37	<0.001
Symptomatic	0.69	0.31 – 1.54	0.362

## Discussion

This retrospective observational study, carried out over a six-week period in 2024, examined the association between PM_2.5_, fine particulate matter, and self-reported asthma symptoms among adults attending a tertiary pulmonology clinic in Dubai, UAE. This is one of the first studies of its kind in this region of the Arabian Peninsula, where the annual average PM_2.5_ concentration is ranked the highest from 2018-2024 data according to IQAir [10]. Unexpectedly, our findings show that higher PM_2.5_ categories (“Unhealthy" and “Unhealthy for sensitive groups”) were associated with a reduced likelihood of reporting SOB, even after adjustments for demographic, lifestyle and clinical cofounders. No statistically significant association was observed between PM_2.5_ levels and cough, wheeze or chest tightness.

The inverse correlation between ambient fine particulate matter and SOB conflicts with previous research, which has consistently demonstrated that particulate matter is an established trigger in asthma exacerbations through mechanisms including airway inflammation and oxidative stress [11]. Even short-term exposure to particulate matter has been seen to be associated with asthma mortality risk [12]. However, several plausible explanations may account for our findings.

Firstly, a major factor that may have contributed to the inverse association between PM_2.5_ and SOB is the reliance on population-level exposure data from outdoor monitoring stations, which is not representative of individual exposure patterns. This is a significant limitation seen in studies assessing air pollution, as this approach fails to capture the actual exposure of study participants, such as time spent indoors, use of air conditioning, commuting, or residential and/or occupational environments. This is particularly relevant in Dubai, where residents spend the majority of their time in air-conditioned environments of varying filtration efficiency. Indoor air pollutants such as SO_2_, NO_2_, and hydrogen sulfide (H_2_S) have been associated with a higher likelihood of wheeze in one study examining the effects of indoor air pollutants and health in the UAE [13]. Additionally, the rate and efficiency of indoor ventilation may affect the concentrations of these indoor pollutants associated with asthma [14].

Another significant aspect is the relatively short six-week observation period in this study. It excluded seasonal variation and dust storm events, which are known to strongly influence particulate matter levels in the region. The inability to capture seasonal representation as a result of the short study duration may be a major reason why our results diverge from the established literature. Importantly, behavioural avoidance has not been accounted for, and it may have affected symptom reporting as asthmatic individuals may have limited their outdoor exposure and deferred non-urgent clinic visits during high pollution days or even adopted protective measures such as indoor air filtration. Moreover, the consultation type may have acted as a confounder, as follow-up visits, which were less likely to involve acute symptom reporting, were more frequent on days with poor air quality. Finally, the relatively small number of observations in the highest and lowest PM_2.5_ categories (“Unhealthy” and “Good” respectively) may have limited statistical power, widened confidence intervals, and thus contributed to the unexpected direction of effect. For instance, the OR for the ‘Unhealthy’ group (0.33, 95% CI: 0.09-1.04) demonstrates how sparse data in this category led to wide confidence intervals and statistical instability. This makes it difficult to determine firm conclusions and should be considered when interpreting the apparent inverse association. All of these factors are particularly relevant as they could be significant reasons for the disparity with existing literature.

This study offers several important strengths that contribute to the literature on asthma and environmental exposure in desert climates. To the best of our knowledge, this is the first study in Dubai to link individual clinician-recorded asthma symptoms with daily air quality data, providing a region-specific perspective and allowing for a more clinically relevant outcome assessment. Symptom data were derived from structured outpatient consultations, reducing recall bias as symptoms were recorded concurrently with the clinical encounter. Furthermore, using logistic regression models to adjust for multiple potential confounders, including demographic and lifestyle variables, further strengthens internal validity.

Limitations and future research

Several limitations should also be acknowledged, with perhaps the most significant being the limited data collection period and lack of accessible environmental data, as previously mentioned. The short six-week observation period in May captures only a narrow seasonal window and may not reflect the influence of longer-term or seasonal variation in PM_2.5_ levels, particularly during peak dust storm months. Current research supports that there are seasonal variations in ambient particulate matter levels. A recent study in Thailand looking at the association of particulate matter on asthma control determined that with every 10 µg/m^3^ increase in the monthly average PM_10_, asthma control test scores significantly decreased [15]. This significant decrease demonstrates the effect of seasonal pollution variation on asthma control, which is something that was not captured in our data collection period. Secondly, and as discussed previously, exposure misclassification is likely, as PM_2.5_ data were sourced from fixed outdoor monitoring stations and may not represent individual exposure, including residential and occupational exposure.

Another limitation was that symptom severity was based on patient self-report during clinical consultation without objective pulmonary function measures such as peak expiratory flow or spirometry values. This limits the ability to confirm the physiological effect of particulate matter and may lead to subjective symptom interpretation between individuals. Additionally, the unit of analysis was consultations rather than individual patients. The dataset included new, symptomatic, and routine follow-up consultations, meaning that some individuals contributed multiple attendances. As a result, the data is not entirely independent, and this clustering at the patient level could introduce bias and may have contributed to the observed inverse association.

Finally, our analysis did not incorporate other potentially relevant environmental variables, including various atmospheric gases, humidity, or temperature, which may act as confounders or effect modifiers. While we chose to focus on the effects of particulate matter due to its established association with deeper airway penetration and inflammation, research has revealed that other outdoor pollutants, such as O_3_, NO_2,_ CO, and SO_2,_ also have negative impacts on asthma outcomes [16].

While the observed inverse association is likely attributable to behavioural and methodological factors rather than a protective effect of pollution, the findings underscore the importance of considering behavioural responses in environmental epidemiology. Our findings could suggest a unique behavioural response in asthmatic individuals living in Dubai in response to poor air quality alerts. For clinical practice, patient education on the use of real-time air quality data to maintain good asthma control remains important. From a public health perspective, the results highlight the need for integrated monitoring systems that combine environmental, behavioural, and clinical data to better capture the true burden of pollution on pulmonology health.

This study serves as a pilot investigation into the relationship between PM_2.5_ and asthma symptoms in the UAE. Future large-scale studies should build upon this framework by employing longer observation periods to capture seasonal variability, incorporating personal exposure monitoring, and including objective measures of lung function. Multi-pollutant models considering CO, SO_2_, O_3_, NO_2_, temperature, and humidity would help characterise the contributions of different environmental exposures. In addition, qualitative and behavioural research could clarify how individuals modify activities and healthcare utilisation in response to poor air quality alerts. Such work would provide a more comprehensive understanding of the true burden of air pollution on asthma in desert climates and inform targeted public health interventions tailored to the UAE’s unique environmental conditions.

## Conclusions

Asthma is a complex chronic condition, with environmental exposures, particularly PM_2.5_, known to play a role in the exacerbation of asthma symptoms. Being the first study to explore patient-reported symptoms with daily air quality data in the UAE, this research was able to provide region-specific insights into the impact of ambient fine particulate matter on asthma. Although our study, suggesting an inverse association between PM_2.5_ and SOB, does not support prior background research, it is important to clarify that this unexpected association should not be interpreted as suggesting a protective role of air pollution. It is more plausibly explained by numerous confounding factors and the short study duration that may have impacted the results. Nevertheless, our study provides valuable insights, paving the way for future studies that should incorporate seasonal variation and a wider spectrum of pollutants. This will allow further assessment of the impact of air pollution exposure on the exacerbation of asthma symptoms prior to extending the implications into clinical practice.
